# The Autobiography of Berzelius

**Published:** 1853-03

**Authors:** 


*The Autobiography of Berzelius.*—After the death of this great Swedish chemist, it was found that he had left behind him a copious and highly interesting biography, for the publication of which we have long and anxiously waited. It appears, however, that the autobiography contains so many personal allusions, and so many unsparing criticisms of living or recently deceased scientific men, that the executors are not only afraid of publishing, but are actually prevented from doing so by the laws of Sweden, which forbid the printing of works dealing in personalities, until several years after the death of their authors. From the known dislike of Berzelius to Sir Humphrey Davy, and to the recently deceased Dr. Thos. Thomson, it is supposed that these eminent chemists figure largely in the suppressed work. We feel sure that the surviving relatives of these distinguished men would prefer the opportunity of answering such criticism themselves, rather than leave the task to a future generation. We trust that the obstacles which prevent the appearance of this—as we are told—remarkable work, may be overcome, and that speedily.—*London Lancet.*

